# Identification of the *MYH6* c.804G>C Synonymous Variant Causing Exon Skipping in a Hypertrophic Cardiomyopathy Family

**DOI:** 10.1002/mgg3.70268

**Published:** 2026-07-08

**Authors:** Songlin Zhang, Xiaohua Tang, Leixiang Yang, Yang Wang, Yong Cui, Xiaopan Chen

**Affiliations:** ^1^ Laboratory Medicine Center, Department of Genetic and Genomic Medicine Zhejiang Provincial People's Hospital, Affiliated People's Hospital, Hangzhou Medical College Hangzhou Zhejiang China; ^2^ Key Laboratory of Birth Defects Wenzhou Central Hospital Wenzhou Zhejiang China; ^3^ Heart Center, Department of Cardiovascular Surgery Zhejiang Provincial People's Hospital, Affiliated People's Hospital, Hangzhou Medical College Hangzhou Zhejiang China

**Keywords:** exon skipping, hypertrophic cardiomyopathy, *MYH6*, synonymous variant

## Abstract

**Background:**

Hypertrophic cardiomyopathy (HCM) is a common hereditary cardiac disorder characterized by left ventricular hypertrophy, outflow tract obstruction, arrhythmias, and increased risk of sudden cardiac death.

**Methods:**

Clinical phenotypes and family histories were collected from affected individuals. Whole‐exome sequencing (WES) followed by Sanger sequencing identified and validated genetic variants. In silico tools predicted splicing effects, with aberrant splicing confirmed by minigene assays.

**Results:**

The proband, a 33‐year‐old male with asymmetric obstructive HCM, exhibited characteristic electrocardiographic, echocardiographic, cardiac MRI, and histopathological findings. WES revealed *MYBPC3* c.787G>A and *MYH6* c.804G>C (p.Leu268=) variants. Segregation analysis showed that only the synonymous *MYH6* variant co‐segregated with HCM in affected family members. This variant was absent from public databases (gnomAD and ClinVar) and the literature as of December 15, 2025. Although three in silico tools predicted negligible splicing impact, minigene assays in HEK293T and HeLa cells revealed partial exon 10 skipping, resulting in an in‐frame deletion (p.Leu268_Asp300del) in approximately 6.8% and 4.7% of transcripts, respectively.

**Conclusion:**

The synonymous *MYH6* c.804G>C variant promotes exon skipping and is associated with HCM. This study provides functional evidence supporting its potential pathogenicity and underscores the value of experimental validation in genetic diagnosis. These findings highlight the limitations of computational predictions and emphasize the importance of functional assays for evaluating synonymous variants in sarcomeric genes.

## Graphical abstract

Although in silico tools predicted minimal splicing impact, functional minigene assays demonstrate that the synonymous *MYH6* c.804G>C variant induces partial exon 10 skipping (~6.8% in HEK293T cells and ~4.7% in HeLa cells), supporting its potential contribution to HCM pathogenesis.

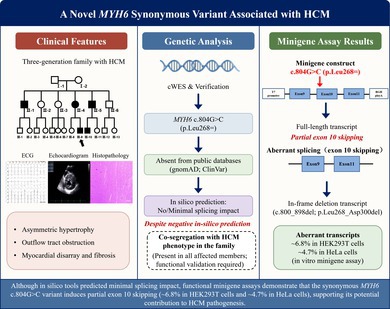

## 1 Introduction

Hypertrophic cardiomyopathy (HCM) is a primary myocardial disorder characterized by asymmetric hypertrophy of the left ventricular myocardium (Lopes et al. 2024). Potential clinical consequences include left ventricular outflow tract obstruction, arrhythmias, sudden cardiac death, heart failure, and myocardial fibrosis (Lee et al. 2022). The etiology of HCM is complex and is usually caused by pathogenic variants in genes encoding sarcomere‐related proteins or other unknown factors. Patients with identifiable genetic mutations account for approximately 40% of HCM cases. Several associated genes have been identified, such as *MYH7*, *MYBPC3*, and *MY*H6 (Anfinson et al. 2022; Liu et al. 2025; Suzuki et al. 2022). Traditionally, the prevalence has been considered 1 in 500; however, emerging evidence suggests that with advances in genetic testing and imaging technologies, the actual prevalence may be higher (Massera et al. 2023). In most age groups, a maximum left ventricular wall thickness of 15 mm at any site is sufficient for a diagnosis of HCM; when the wall thickness is 13 to 14 mm, diagnosis can also be made if there is a family history of HCM, typical dynamic outflow tract obstruction, or distinctly abnormal electrocardiographic patterns (Rowin et al. 2018; Maron et al. 2022).

Cardiac myosin is a principal structural and functional constituent of the sarcomere, the fundamental contractile unit of cardiac muscle (Toepfer et al. 2020). The *MYH6* gene encodes the α‐myosin heavy chain (α‐MHC), a fast ATPase isoform predominantly expressed in the atrial myocardium, and its mutations have been associated with HCM (Anfinson et al. 2022). In the adult human heart, myosin heavy chains in the ventricles are composed of approximately 95% β‐ and 5% α‐isoforms, whereas in the atria the ratio is roughly 5% β‐ to 95% α‐, with the β‐isoform encoded by the *MYH7* gene (β‐MHC) (Anfinson et al. 2022). The *MYH6* gene, located at 14q11.2, spans 26,159 bp and includes a 4484‐bp 5′ flanking intergenic region. It consists of 39 exons, 37 of which are protein‐coding (Marian 2021). A large‐scale genetics study found rare *MYH6* variants in about 3.8% of HCM patients (Walsh et al. 2017). However, because *MYH6* also has a relatively high background variant rate in the general population, its association with HCM is considered only “weak evidence”. Compared with major disease‐causing genes like *MYH7* and *MYBPC3*, the clinical significance of *MYH6* variants is more difficult to interpret (Ingles et al. 2019).

Mutations are permanent alterations in the DNA sequence and are typically classified as nonsynonymous (which alter the encoded amino acid sequence) or synonymous (which do not change the protein's primary structure). Due to their “silent” nature, synonymous mutations were long considered neutral or nearly neutral evolutionary markers. However, accumulating evidence has fundamentally challenged this view, demonstrating that synonymous mutations can influence virtually every stage of gene expression—from transcription and mRNA processing to translation and co‐translational protein folding—often exerting significant non‐neutral effects on organismal fitness (Sauna and Kimchi‐Sarfaty 2022; Zhang and Qian 2025). Mechanistically, synonymous substitutions can modulate translational elongation speed and accuracy by altering codon usage bias, affect mRNA stability and secondary structure, or disrupt splicing signals and other cis‐regulatory elements embedded within exons. Large‐scale analyses of human cancer genomes have revealed that a substantial proportion of synonymous mutations function as driver mutations, with estimates ranging from 20% to 50% in oncogenes, accounting for approximately 6%–8% of all selected single‐nucleotide variants. These mutations frequently alter splicing regulatory motifs, such as exonic splicing enhancers (ESEs) (Supek et al. 2014). Notably, synonymous mutations can interfere with splice site recognition or exonic splicing enhancer elements, leading to exon skipping, intron retention, or the production of aberrant splice isoforms. Such changes subsequently influence mRNA secondary structure, splicing factor binding, translational kinetics, and overall mRNA stability (Chu and Wei 2019). In clinical contexts, for instance, synonymous mutations in the *MYH7* gene have been shown to cause HCM by inducing pathogenic exon skipping (Surikova et al. 2019). Collectively, two decades of research have established that synonymous mutations are far from functionally silent. Instead, they can perturb multiple layers of the central dogma, including post‐transcriptional regulation, splicing fidelity, and translational efficiency, thereby contributing to disease pathogenesis. These findings underscore the need for disease mechanism studies to move beyond the amino acid sequence and incorporate nucleotide‐level and codon‐based variation, with careful consideration of their regulatory consequences on RNA processing and protein homeostasis.

The contribution of synonymous mutations is often underestimated, which may lead to missed diagnoses in some cases. Research on *MYH6* synonymous mutations remains limited, and their pathogenic mechanisms are not yet fully understood. Minigene assays represent an important in vitro approach for validating splicing variants and are valuable for assessing the functional impact of synonymous mutations. In this study, we analyzed the clinical phenotype and genetic characteristics of an HCM family carrying the *MYH6* p.Leu268 = synonymous mutation. By integrating Minigene experiments, bioinformatic analyses, and evolutionary conservation assessments, we aimed to elucidate the pathogenicity of this variant and provide evidence for precise HCM diagnosis and genetic counseling.

## 2 Materials and Methods

### 2.1 Ethical Compliance

This study was approved by the Medical Ethics Committee of Zhejiang Provincial People's Hospital (approval number: Zhejiang Provincial Medical Ethics Review 2025 Other No. 244). Written informed consent was obtained from the proband for study participation, genetic testing, and publication of this case report, including detailed clinical data, electrocardiographic images, echocardiographic images, cardiac MRI findings, histopathological images, and the family pedigree chart. All study procedures were conducted in accordance with the ethical principles of the Declaration of Helsinki.

### 2.2 Clinical Data

Clinical data were collected from a 33‐year‐old male patient who was referred to Zhejiang Provincial People's Hospital with a clinical suspicion of HCM. The evaluation included standard 12‐lead electrocardiography, transthoracic echocardiography, and other comprehensive cardiac assessments. After confirmation of hypertrophic obstructive cardiomyopathy, the patient underwent thoracoscopic left ventricular outflow tract myectomy combined with mitral valve repair. Myocardial tissue obtained from the surgical margins was subjected to histopathological examination.

### 2.3 Pedigree Investigation and Sample Collection

**FIGURE 1:**
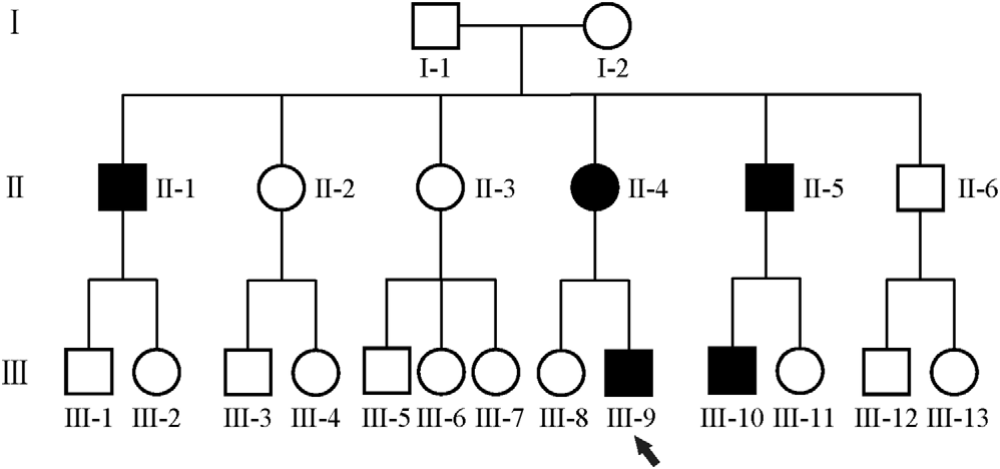
Family pedigree chart. The square represents males, the circle represents females, the black‐filled area represents HCM patients, the blank area represents normal, and the arrow points to the proband.

A detailed three‐generation pedigree investigation was conducted for the proband and participating relatives, including direct descendants and collateral lines. Sex, age, clinical status, and available HCM‐related phenotypic information were recorded. The pedigree is presented in Figure 1.

Peripheral venous blood samples (5 mL, EDTA‐anticoagulated) or saliva samples were collected from the proband and seven additional family members. Genomic DNA was extracted and shipped on dry ice to the Department of Genetic and Genomic Medicine, Zhejiang Provincial People's Hospital, for sequencing and analysis. Control samples were obtained from unrelated healthy individuals using identical collection methods.

### 2.4 Family‐Based WES and Validation

Peripheral blood from the proband was collected in EDTA tubes, and genomic DNA was extracted using the QIAamp DNA Blood Mini Kit (QIAGEN, Germany). DNA concentration and purity were assessed using a NanoDrop 2000 spectrophotometer and a Qubit 3.0 fluorometer (Thermo Fisher Scientific, USA). Whole‐exome sequencing (WES) was performed on the Illumina NextSeq 550 platform (Illumina, USA) using the SureSelect Human All Exon V7 capture kit (Agilent Technologies, USA). Sequencing reads were aligned to the human reference genome GRCh37/hg19 using the Burrows‐Wheeler Aligner (BWA‐MEM, v0.7.17). Subsequent data processing, variant calling, and quality control followed the Genome Analysis Toolkit (GATK, v4.2.0.0) best practices workflow for germline short variant discovery.

Variants with a minor allele frequency (MAF) < 0.1% were retained, based on population databases including the 1000 Genomes Project, ExAC, gnomAD, and ESP6500. In silico splicing impact predictions were performed using SpliceAI. Candidate variants were classified according to the American College of Medical Genetics and Genomics/Association for Molecular Pathology (ACMG/AMP) guidelines.

All candidate pathogenic or likely pathogenic variants were validated by bidirectional Sanger sequencing. Primers were designed using Primer3 (based on GRCh37/hg19). PCR amplification was performed with 2× Taq Master Mix (Vazyme, Nanjing, China), and sequencing was conducted on an ABI 3730xl DNA Analyzer (Applied Biosystems, Foster City, CA, USA). Sequence traces were aligned to the reference genome to confirm the variants.

### 2.5 Minigene Assay

To evaluate the effect of the *MYH6* variant on pre‐mRNA splicing, genomic DNA from the proband and healthy controls was used as a template to amplify the *MYH6* fragment spanning Exon 9–Intron 9–Exon 10–Intron 10–Exon 11 using specific primers (Table S1). PCR products were purified via 1.5% agarose gel electrophoresis, verified, and inserted into the pcDNA3.1 vector to construct wild‐type (wt) and mutant (mut) minigenes. Reagents and instruments used in the experiments are listed in Tables S2 and S3.

#### 2.5.1 Plasmid Construction

Nested primers (2991‐F/4965‐R and 3234‐F/4731‐R) were used to amplify the target fragment from healthy control gDNA (wt) and proband gDNA (mut) (Table S1). The second‐round nested PCR product served as a template for amplification with MYH6‐pcDNA3.1‐KpnI‐F and MYH6‐pcDNA3.1‐XhoI‐R primers to generate the 1105 bp MYH6‐pcDNA3.1‐wt fragment. PCR products were analyzed by gel electrophoresis and purified.

For the mutant construct, the left fragment (365 bp) was amplified using MYH6‐pcDNA3.1‐KpnI‐F and MYH6‐mut‐R primers, and the right fragment (771 bp) was amplified using MYH6‐mut‐F and MYH6‐pcDNA3.1‐XhoI‐R primers. Equal amounts of left and right fragments were mixed and amplified with MYH6‐pcDNA3.1‐KpnI‐F and MYH6‐pcDNA3.1‐XhoI‐R primers to generate the full‐length 1105 bp MYH6‐pcDNA3.1‐mut fragment.

The fragments and pcDNA3.1 vector were digested, purified, ligated, and transformed into DH5α competent cells. Colony PCR and Sanger sequencing were performed to verify the correct insertion. Enzyme digestion, ligation, and culture conditions were carried out according to standard protocols.

#### 2.5.2 Cell Transfection

HeLa and HEK293T cells were obtained from Shanghai Fujun Gene Biotechnology Co. Ltd. (Shanghai, China). HeLa cells were cultured in Dulbecco's Modified Eagle Medium (DMEM) supplemented with 10% fetal bovine serum (FBS). HEK293T cells were cultured in DMEM supplemented with 10% FBS and 1% penicillin–streptomycin. All cells were maintained at 37°C in a humidified incubator with 5% CO_2_. Transfection was performed using Lipofectamine 3000 (Thermo Fisher Scientific) according to the manufacturer's instructions. The culture medium was replaced every 2–3 days, and cells were passaged at approximately 80%–90% confluence using 0.25% trypsin–EDTA.

For transfection experiments, HeLa cells were seeded at a density of 2.0 × 10^5^ cells per well and HEK293T cells at 2.5 × 10^5^ cells per well in six‐well plates, 24 h before transfection with Lipofectamine 3000. Cell numbers were determined using a hemocytometer to ensure consistency across independent experiments.

Wild‐type and mutant recombinant constructs were transiently transfected into HeLa and HEK293T cells using a lipid‐based transfection reagent according to the manufacturer's instructions. Cells were harvested 48 h post‐transfection.

#### 2.5.3 Minigene Transcription Analysis

Total RNA was extracted from transfected HeLa and HEK293T cells using RNAiso PLUS (TaKaRa) according to the manufacturer's instructions. RNA concentration and purity (A260/A280) were measured using a NanoDrop spectrophotometer. Equal amounts of total RNA were reverse‐transcribed into cDNA using ABScript III RT Master Mix with gDNA Remover (ABclonal) following the manufacturer's protocol.

PCR amplification of the pcDNA3.1‐wt/mut minigene transcripts was performed using primers pcDNA3.1‐F and pcDNA3.1‐R (sequences provided in Table S3). PCR products were separated by 1.5% agarose gel electrophoresis. Individual bands were excised, purified, and subjected to Sanger sequencing to determine splicing patterns.

Band intensities on agarose gels were quantified by densitometry using ImageJ software (version 1.53). The proportion of the exon 10‐skipped transcript (band b) was calculated as follows: (intensity of band b)/(intensity of band a + intensity of band b) × 100. Quantification was performed on representative gels from the transfection experiments in HEK293T and HeLa cells.

### 2.6 Splicing Prediction

To assess the presumptive effect of this variant on splicing, three different in silico prediction tools: SpliceAI (https://spliceailookup.broadinstitute.org), RNA Splicing Prediction Model (https://rddc.tsinghua‐gd.org/zh/tool/rna‐splicer), and Human Splicing Finder Version (https://hsf.genomnis.com/mutation/analysis) were used.

## 3 Results

### 3.1 Clinical Presentation of the Proband

The proband was a 33‐year‐old male who presented with a history of exertional chest tightness and palpitations for over 1 year. He was admitted to Zhejiang Provincial People's Hospital on September 27, 2022, with a clinical suspicion of HCM, and subsequently underwent comprehensive evaluation and management in the Department of Cardiovascular Surgery. Clinical data were retrospectively collected.

**FIGURE 2:**
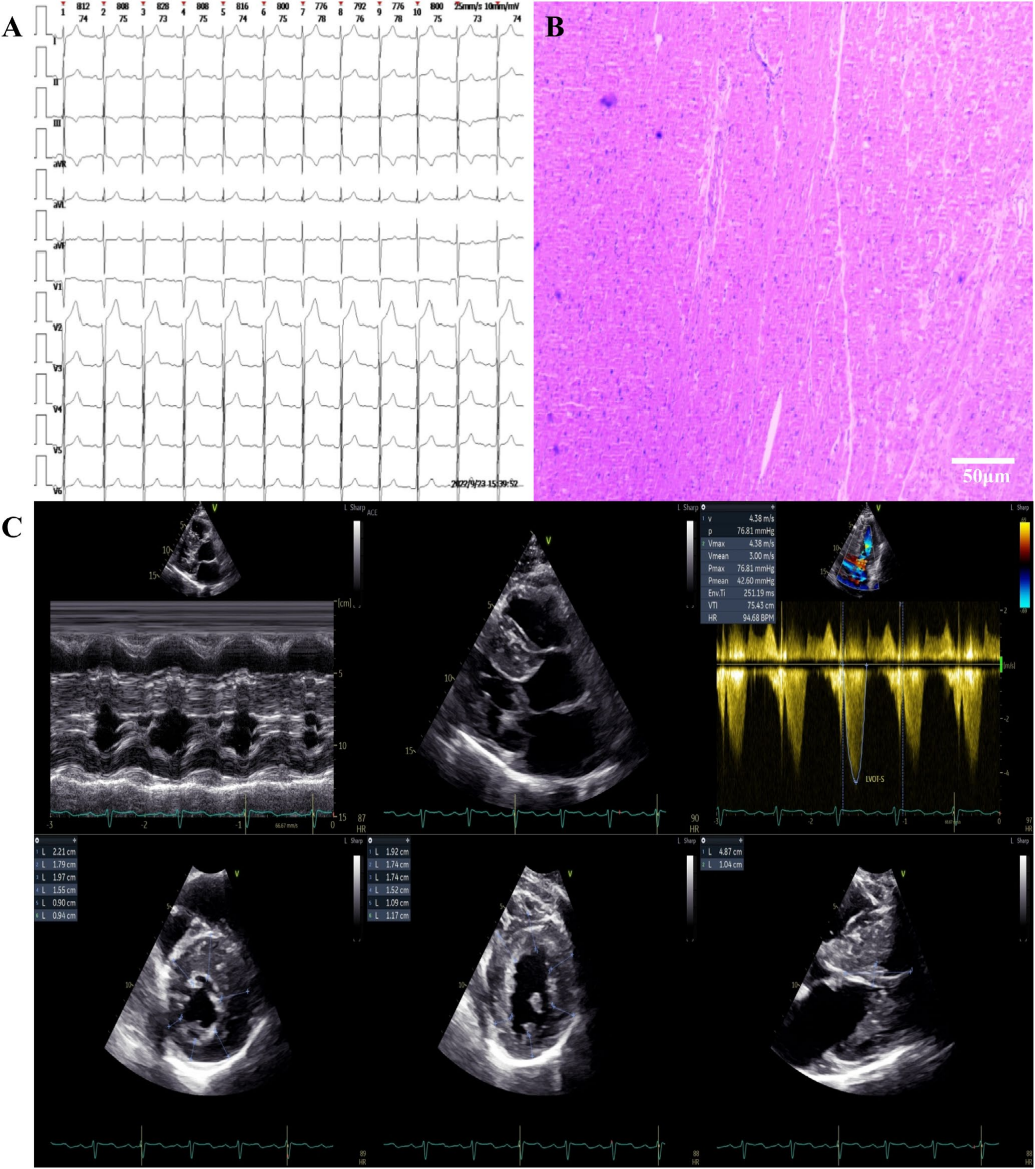
Key diagnostic findings in the proband. (A) Electrocardiogram: Sinus rhythm with right‐axis deviation. (B) Myocardial histopathology from surgical margin: Cardiomyocyte hypertrophy, fiber disarray, endocardial thickening, and interstitial fibrosis (gross specimen size ≈3.5 × 2.5 × 1 cm). (C) Echocardiography: Asymmetric septal hypertrophy (max 21 mm), LVOT obstruction (PSV 4.4 m/s, PGmax 77 mmHg), SAM with mild‐to‐moderate mitral regurgitation, mild left atrial enlargement (44 × 49 mm), small LV cavity (EDV 77 mL, SV 60 mL), and diastolic dysfunction (e′ < a′).

A thorough cardiac evaluation was performed, including 12‐lead electrocardiography, transthoracic echocardiography, and cardiac magnetic resonance imaging (CMR). Electrocardiography revealed a sinus rhythm with right‐axis deviation (Figure 2A). Transthoracic echocardiography demonstrated asymmetric obstructive HCM, characterized by significant hypertrophy predominantly involving the basal to mid interventricular septum and anterior left ventricular wall (maximal wall thickness 21–22 mm). The left ventricular cavity was relatively small, with abnormal hypertrophic muscle bundles extending from the basal septum toward the apical anterior wall, resulting in mid‐cavity narrowing (Figure 2C). Systolic anterior motion (SAM) of the mitral valve was present, accompanied by left ventricular outflow tract (LVOT) obstruction (peak systolic velocity ≈4.4 m/s, maximal pressure gradient ≈77 mmHg), elongated mitral valve leaflets with abnormal secondary chordae tendineae, and mild‐to‐moderate eccentric mitral regurgitation. Mild left atrial enlargement was observed (44 × 49 mm), with preserved left ventricular systolic function and grade 1–2 diastolic dysfunction. Histopathological examination of myocardial tissue from the surgical margin (gross appearance: two gray‐yellow to gray‐white solid tissue pieces, total size ≈3.5 × 2.5 × 1 cm) showed classic features of HCM, including marked cardiomyocyte hypertrophy, myocardial fiber disarray, focal endocardial thickening, and interstitial fibrosis, consistent with chronic myocardial remodeling under abnormal hemodynamic load or genetic influence (Figure 2B).

Based on the above clinical, electrocardiographic, echocardiographic, imaging, and pathological findings, the patient was diagnosed with asymmetric obstructive HCM in accordance with the 2020 AHA/ACC Guideline for the Diagnosis and Treatment of Patients With HCM (Ommen et al. 2020).

### 3.2 Family Investigation

The pedigree spanned three generations with 21 individuals, of whom five (II‐1, II‐4, II‐5, III‐9, III‐10) were diagnosed with HCM, showing maternal‐line segregation. No consanguinity was reported. Affected relatives included the proband's mother (septal thickness 18 mm), maternal uncle (15 mm), younger maternal uncle (22 mm), and the son of the younger maternal uncle (25 mm). The proband's elder sister exhibited impaired left ventricular diastolic dysfunction with normal septal thickness. Phenotypes in affected individuals were characterized by septal hypertrophy (range 15–25 mm), obstructive features, and/or arrhythmias. II‐5 was diagnosed with HCM based on clinical evaluation but declined genetic testing. The pedigree is shown in Figure 1.

### 3.3 WES Analysis

WES of the proband identified heterozygous variants: *MYBPC3* (NM_000256.3) c.787G>A (p.Gly263Arg) and *MYH6* (NM_002471.3) c.804G>C (p.Leu268=). Sanger sequencing validated these findings, and segregation analysis was performed for both.

For the *MYBPC3* c.787G>A variant, only the proband carried it, while other family members were wild‐type, indicating no segregation with the phenotype. Bioinformatic analysis indicated that this missense variant changes glycine to arginine at codon 263. According to ACMG guidelines and database information, this variant was classified as a variant of uncertain significance (VUS) (Figure S1).

**FIGURE 3:**
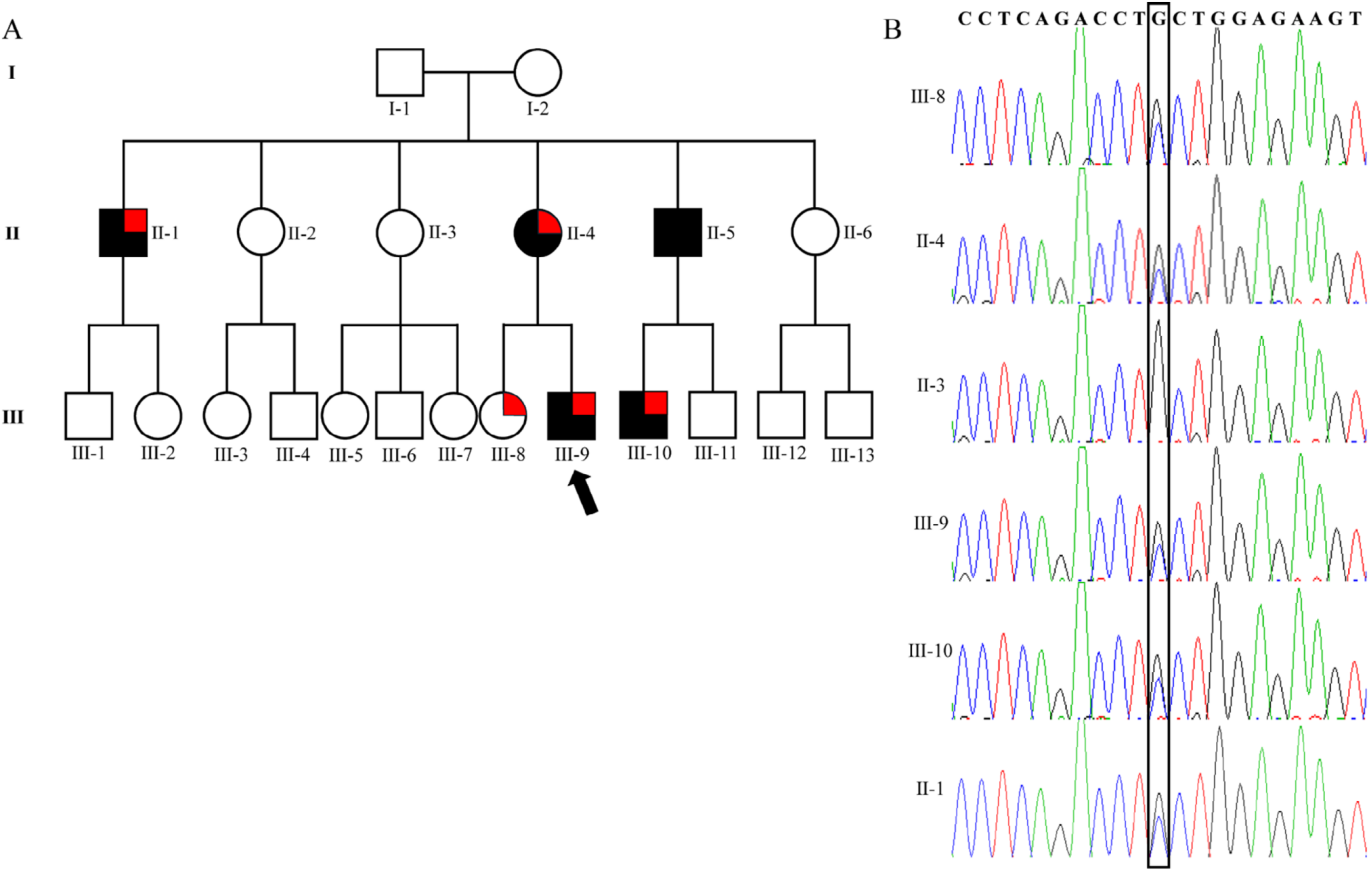
WES analysis and Sanger sequencing validation results for the *MYH6* c.804G>C variant. (A) Pedigree of the HCM family. Affected individuals are indicated by filled symbols. The proband (III‐9) is marked with an arrow. (B) Sanger sequencing chromatograms showing the *MYH6* c.804G>C variant. The variant is present in the tested affected members II‐1, II‐4, III‐8, III‐9, and III‐10 but absent in the unaffected individual II‐3. Note that individual II‐5 was clinically diagnosed with HCM but declined genetic testing and was therefore not included in Sanger sequencing.

For the *MYH6* c.804G>C variant, Sanger sequencing validation was performed in six family members (II‐1, II‐3, II‐4, III‐8, III‐9, III‐10). The variant was detected in all tested affected individuals (II‐1, II‐4, III‐8, III‐9, III‐10), but was absent in the unaffected individual II‐3. This result supports the co‐segregation of the variant with the HCM phenotype in the tested family members. A literature search up to December 2025 revealed no prior reports of this variant in public databases or publications, confirming it as novel. Bioinformatic analysis indicates that *MYH6* c.804G>C is a synonymous variant that does not alter the encoded amino acid. According to the ACMG/AMP guidelines, this variant was classified as a variant of uncertain significance (VUS) (Figure 3).

### 3.4 Minigene Splicing Assay

**FIGURE 4:**
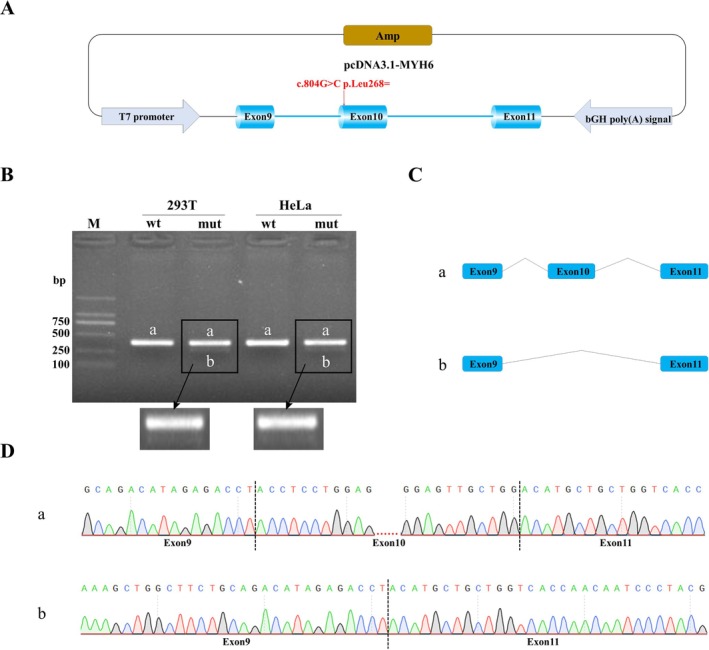
Detection results of the pcDNA3.1 minigene constructs. (A) Schematic diagram of minigene vector construction. (B) RT‐PCR analysis of splicing patterns in 293T and HeLa cells. Mut, mutant minigene; WT, wild‐type minigene. Band a: Normal splicing product (Exon9–Exon10–Exon11); Band b: Exon 10‐skipped product (Exon9–Exon11). (C) Schematic diagram of normal and aberrant splicing. (D) Sanger sequencing confirmation of the splicing products. The proportion of the exon 10‐skipped transcript in the mutant was estimated by densitometry analysis of gel band intensities using ImageJ (version 1.53) on the representative gel shown. The proportion of exon 10‐skipped transcripts was quantified by densitometry on representative gels and calculated as approximately 6.8% in HEK293T cells and 4.7% in HeLa cells.

To assess the splicing impact of *MYH6* c.804G>C, wild‐type and mutant minigene constructs were generated and transfected into HeLa and 293 T cells (Figure 4A,B). Plasmid verification through sequencing confirmed the correct insertion in the minigene constructs. Total RNA extracted from transfected cells showed concentrations and purity within acceptable ranges (Table 1).

**TABLE 1:** RNA concentration and purity of minigene‐transfected cells.

Sample ID	Concentration (ng/μL)	Purity (A260/A280)
293T‐wt	923	1.931
293T‐mut	867	1.862
HeLa‐wt	981	1.972
HeLa‐mut	944	1.945

RT‐PCR and agarose gel electrophoresis revealed that the wild‐type minigene produced a single band of the expected size (374 bp, band a), corresponding to normal splicing of Exon9–Exon10–Exon11. In contrast, the mutant minigene produced two bands: band a (normal splicing) and a smaller band b (Figure 4B). A schematic depicting these normal and aberrant splicing patterns is presented in Figure 4C. Sanger sequencing confirmed that band b represented the exon 10‐skipped transcript (Exon9–Exon11) (Figure 4D). Densitometry analysis of representative gels using ImageJ software showed that the exon 10‐skipped transcript (band b) constituted approximately 6.8% of total transcripts in HEK293T cells and 4.7% in HeLa cells. The wild‐type minigene produced only the full‐length transcript (band a) in both cell lines. The predicted protein consequence of this exon 10 skipping is an in‐frame deletion, c.800_898del (p.Leu268_Asp300del), resulting in an internal deletion of 33 amino acids without frameshift, generating an internally deleted protein of 1906 amino acids (Figure 4D).

### 3.5 Splice Mutation Prediction

In silico splicing predictions were performed for the synonymous variant c.804G>C in *MYH6* (MANE Select transcript, negative strand) using multiple tools. SpliceAI analysis yielded very low delta scores: acceptor gain (AG) = 0 (position −692 bp), acceptor loss (AL) = 0.01 (position + 150 bp), donor gain (DG) = 0 (position +100 bp), and donor loss (DL) = 0.01 (position −94 bp), with a maximum Δ score of 0.01 (Table S4). The RNA Splicing Prediction Model, integrating DanQ and SPTransformer models, evaluated splice site strengths near junctions between exons E9‐E10 and E10‐E11, revealing minimal changes between reference and alternate sequences: DanQ scores of 0.9956 → 0.9956, 1 → 1, 0.8627 → 0.8594, and 0.9997 → 0.9997; SPTransformer scores of 0.9913 → 0.9919, 0.9962 → 0.9958, 0.8241 → 0.8106, and 0.9931 → 0.993; and sequence specificity scores of 0.002, 0.001, 0.014, and 0.001, respectively (Table S5). Human Splicing Finder Pro detected no creation of new splicing signals and reported no significant impact on existing splicing signals (No Signal Interpretation: No significant impact on splicing signals), with the associated transcript ENST00000405093 (Table S6).

## 4 Discussion

This study employed WES to identify a *MYH6* gene c.804G>C synonymous variant in a three‐generation family with HCM. The variant co‐segregated with the disease phenotype and was further functionally evaluated using a minigene splicing assay. Rather than establishing *MYH6* as a major HCM gene, this work provides mechanistic insight into how synonymous variants may affect pre‐mRNA splicing.

Although the proportion of aberrant transcripts (~6.8%) appears modest, several factors should be considered when evaluating its potential biological impact. First, *MYH6* represents only a minor fraction (~5%) of ventricular myosin in adult hearts; thus, even a small proportion of aberrant transcripts may constitute a meaningful fraction of the total *MYH6* pool. Second, sarcomeric proteins are highly dosage‐sensitive, and even subtle perturbations may disrupt sarcomere assembly and function. Third, the minigene assay represents an artificial system that lacks the full complement of cardiomyocyte‐specific splicing factors and chromatin context and therefore may not fully recapitulate endogenous splicing regulation. Taken together, these considerations raise the possibility that even a relatively low level of aberrant splicing could have functional consequences. *MYH6* is not considered a core HCM gene, and its gene–disease association remains less well established compared with canonical sarcomeric genes such as *MYH7* and *MYBPC3*. Notably, previous studies have shown that even low levels of aberrant splicing can contribute to disease pathogenesis in cardiomyopathy‐related genes. Alternatively, it is also possible that this level of exon skipping is insufficient to produce a clinically significant effect, particularly given the relatively low expression of *MYH6* in ventricular myocardium.

The experimental results from the minigene splicing assay demonstrated that the *MYH6* c.804G>C variant induces skipping of exon 10, resulting in an in‐frame deletion of 33 amino acids and production of an internally deleted protein. In the representative minigene assay, the exon 10‐skipped transcript constituted approximately 6.8% of the total *MYH6* transcripts in HEK293T cells and 4.7% in HeLa cells. In contrast, the study also identified a *MYBPC3* c.787G>A missense variant that was present only in the proband and did not cosegregate with the HCM phenotype across the family. Bioinformatics analysis classified this *MYBPC3* variant as a VUS, suggesting it is more likely an incidental finding or one with low penetrance rather than the primary driver of disease in this pedigree (Figure S1).

Multiple sequence alignment demonstrated that the *MYH6* c.804G>C variant site is highly conserved across humans and other species, underscoring its functional importance. *MYH6* encodes α‐myosin heavy chain (α‐MHC), a critical structural component of the myocardial contractile apparatus that plays an essential role in left ventricular contractile dynamics. Although the *MYH6* c.804G>C variant is synonymous, three independent in silico splicing prediction tools consistently indicated no substantial disruption to canonical pre‐mRNA splicing mechanisms: SpliceAI yielded very low delta scores (maximum Δ = 0.01), well below thresholds typically associated with splicing pathogenicity (Table S4); the RDDC RNA Splicer showed only minimal alterations in splice‐site probabilities near the E9–E10 and E10–E11 junctions, with sequence specificity scores ranging from 0.001 to 0.014 (Table S5); and Human Splicing Finder Pro detected neither the creation of new splicing signals nor any significant impact on existing ones (Table S6). These concordant negative predictions from methodologically diverse in silico tools—encompassing probabilistic delta‐score modeling (SpliceAI), transformer‐based architectures (SPTransformer), and traditional motif‐ and matrix‐based approaches (HSF Pro)—indicate that the *MYH6* c.804G>C variant does not impair recognition of canonical splice sites or activate cryptic splice sites (Jaganathan et al. 2019; Ribeiro et al. 2020). Consequently, the minigene findings suggest that the variant may affect non‐canonical splicing regulatory elements rather than directly disrupting canonical splice donor or acceptor sites. Instead, we hypothesize that the variant exerts its effect through more subtle regulatory mechanisms, such as alteration of exonic splicing enhancers or silencers (ESE/ESS), modification of RNA secondary structure, or interference with splicing factor recruitment and co‐transcriptional processing. The resulting in‐frame deletion (exon 10 skipping) removes a highly conserved segment within the myosin head domain that is essential for actin interaction and force generation, thereby providing a plausible molecular explanation for the impaired myocardial contractility and hypertrophic phenotype observed in affected individuals.

Notably, synonymous variants are frequently underestimated in pathogenicity assessments because they do not alter the amino acid sequence (Singer et al. 2023). However, this study suggests that the c.804G>C variant may disrupt pre‐mRNA splicing, leading to exon skipping—a mechanism that has been reported in cardiomyopathy‐related genes but documented in other genetic disorders, such as *TTN* synonymous variants in dilated cardiomyopathy and *FBN1* synonymous variants in Marfan syndrome (Li et al. 2020; Wu et al. 2025). These findings further underscore the critical importance of functional validation experiments in evaluating the pathogenicity of synonymous variants. Furthermore, the variant does not introduce a frameshift or premature termination codon (PTC), making nonsense‐mediated mRNA decay (NMD) unlikely. One possible pathogenic mechanism may involve a dominant‐negative effect, whereby the altered α‐MHC protein interferes with normal sarcomere function. However, this hypothesis requires further experimental validation. At present, no protein‐level or functional assays have been performed to confirm this mechanism.

The strengths and highlights of this study include the comprehensive integration of multi‐platform sequencing and functional validation, achieving a complete research cycle from genetic detection to mechanistic verification. The use of WES maximized variant discovery coverage and accuracy, providing robust technical support for identifying rare variants. The minigene assay directly confirmed the splicing effect, offering functional evidence supporting a possible contribution of this synonymous variant. Furthermore, the study provided supportive evidence for the potential pathogenic relevance of *MYH6* c.804G>C through family cosegregation analysis, population frequency databases, conservation analysis, structural predictions, and bioinformatics assessments, providing new insights into the molecular diagnosis of HCM. The proposed dominant‐negative effect as a potential pathogenic mechanism offers a novel perspective for investigating the functional impact of synonymous variants and broadens our understanding of HCM pathogenesis. The discordance between in silico predictions and experimental findings highlights the limitations of current splicing prediction tools, particularly for non‐canonical splice‐altering variants.

Nevertheless, the study has certain limitations. Although the minigene assay effectively demonstrated the splicing defect, as an in vitro model, it cannot fully recapitulate the complex splicing regulatory environment in cardiomyocytes, potentially leading to under‐ or overestimation of the variant's actual impact. The relatively small family size limits the statistical power of cosegregation analysis, necessitating validation in additional families or large‐scale cohorts to confirm the variant's frequency distribution and pathogenicity. Additionally, the absence of in vivo functional validation—such as in animal models or induced pluripotent stem cell (iPSC)‐derived cardiomyocytes—restricts direct assessment of the variant's effects on myosin function and myocardial contractility. Although the *MYBPC3* c.787G>A variant did not cosegregate with the phenotype, its potential role as a modifier cannot be entirely excluded and warrants further investigation. In conclusion, this study describes a novel *MYH6* c.804G>C synonymous variant in a family with HCM and provides functional evidence of its effect on mRNA splicing. These findings expand the mutational spectrum of *MYH6* and suggest a potential role for synonymous variants in cardiomyopathy. Further in vivo studies and larger cohorts are required to clarify its pathogenic relevance and clinical significance.

## 5 Conclusion

The *MYH6* c.804G>C synonymous variant is associated with HCM in this family and promotes partial exon 10 skipping, as demonstrated by minigene splicing assays. These findings suggest a potential pathogenic mechanism involving aberrant splicing; however, further studies are required to determine its functional and clinical significance. As next‐generation sequencing becomes more widely used in the diagnosis of genetic disorders, synonymous variants are increasingly being identified. This study adds to the growing body of evidence highlighting the importance of carefully evaluating synonymous variants in sarcomeric genes and supports the value of functional assays, such as minigene or cDNA analysis, in assessing their potential pathogenicity.

## Author Contributions

S.Z., Y.C., and X.C. conceived and designed the study. S.Z., X.T., and L.Y. performed the experiments and data analysis. Y.W. contributed to sample collection and clinical data preparation. S.Z. drafted the manuscript. Y.C. and X.C. critically revised the manuscript. All authors discussed the results and approved the final manuscript.

## Funding

This work was supported by grants from the Research Program for Medicine and Health of Zhejiang Province [2025HY0131 (Xiaopan Chen)], the National Science and Technology Major Project of the Ministry of Science and Technology of China [2024ZD0527203‐5 (Yong Cui)], the Joint Science and Technology Plan Project co‐built by the National Administration of Traditional Chinese Medicine and the Administration of Traditional Chinese Medicine of Zhejiang Province [GZY‐ZJ‐KJ‐23046 (Yong Cui)].

## Ethics Statement

This study was approved by the Medical Ethics Committee of Zhejiang Provincial People's Hospital (approval number: Zhejiang Provincial Medical Ethics Review 2025 Other No. 244). Written informed consent was obtained from all participating family members.

## Conflicts of Interest

The authors declare no conflicts of interest.

## Supporting information


**Figure S1:** WES Analysis and Sanger Sequencing Validation Results for the *MYBPC3* c.787G>A Variant. (A) WES analysis revealed that among all affected family members (II‐1, II‐4, II‐5, III‐8, III‐9, and III‐10), only the proband (III‐9) carries the *MYBPC3* c.787G>A variant. (B) Sanger sequencing confirmed that the *MYBPC3* c.787G>A variant is present exclusively in the proband (III‐9), while all other tested individuals (II‐4, III‐9, II‐5, III‐10, II‐1, II‐3, and II‐2) are wild‐type.

**Table S1:** Primers used in the minigene assay.

**Table S2:** Kits and reagents used in this study.

**Table S3:** Instruments used in this study.

**Table S4:** SpliceAI and Pangolin prediction results for the *MYH6* c.804G>C variant.

**Table S5:** RNA splicing prediction model results for splice sites near the *MYH6* variant.

**Table S6:** Human Splicing Finder analysis results.

## Data Availability

The data that support the findings of this study are available from the corresponding author upon reasonable request.
