# Supplementary material for: Identification of the MYH6 c.804G>C Synonymous Variant Causing Exon Skipping in a Hypertrophic Cardiomyopathy Family
Source: Mol Genet Genomic Med. 2026 Jul 8;14(7):e70268. doi: 10.1002/mgg3.70268 (PMC13344885; doi:10.1002/mgg3.70268)
Supplement: Supplementary file 1 — Figure S1: WES Analysis and Sanger Sequencing Validation Results for the MYBPC3 c.787G>A Variant. (A) WES analysis revealed that among all affected family members (II‐1, II‐4, II‐5, III‐8, III‐9, and III‐10), only the proband (III‐9) carries the MYBPC3 c.787G>A variant. (B) Sanger sequencing confirmed that the MYBPC3 c.787G>A variant is present exclusively in the proband (III‐9), while all other tested individuals (II‐4, III‐9, II‐5, III‐10, II‐1, II‐3, and II‐2) are wild‐type. [file MGG3-14-e70268-s007.docx]

**
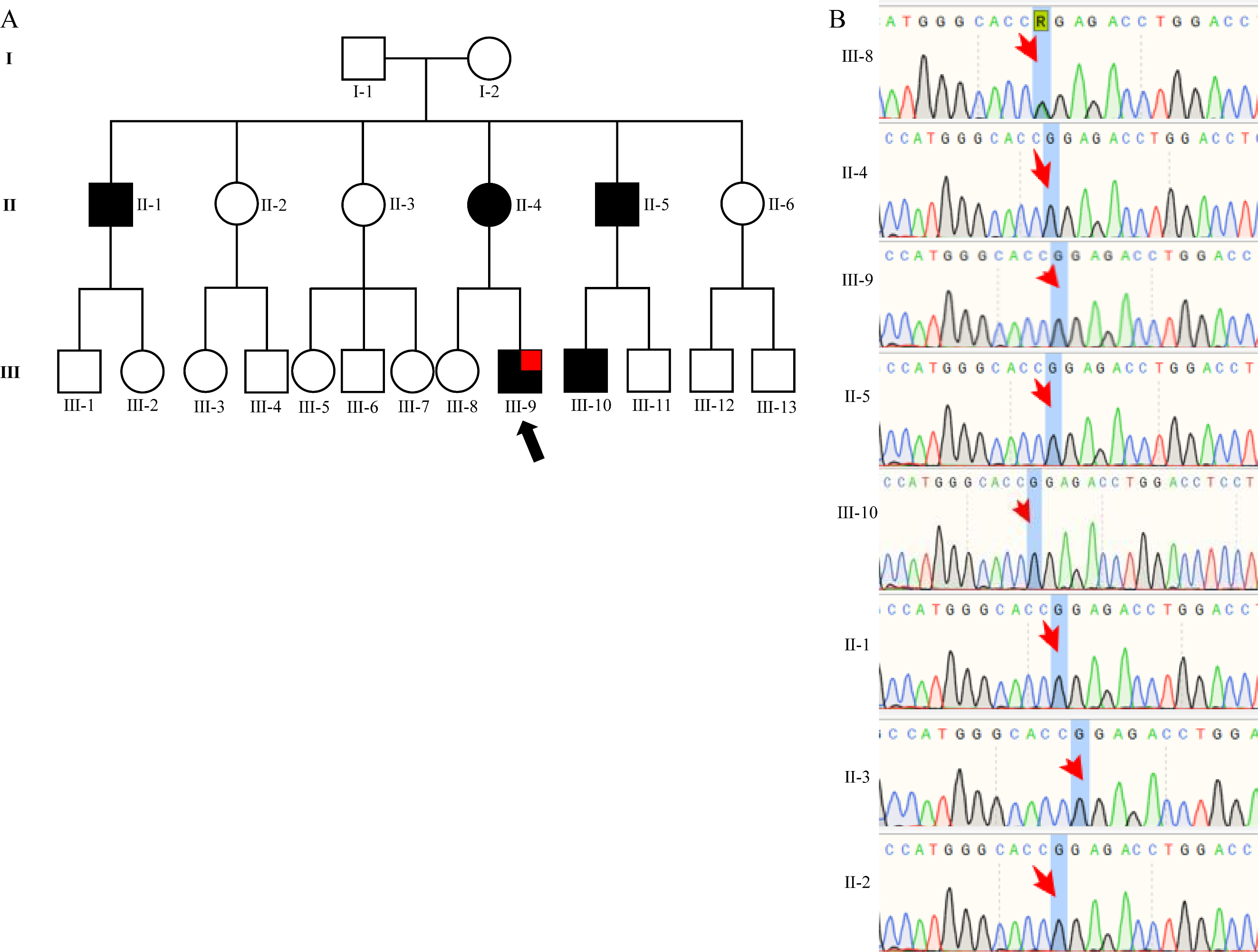
**

**Supplementary Figure 1** | WES Analysis and Sanger Sequencing Validation Results for the *MYBPC3* c.787G>A Variant. (A) WES analysis revealed that among all affected family members (II-1, II-4, II-5, III-8, III-9, and III-10), only the proband (III-9) carries the *MYBPC3* c.787G>A variant. (B) Sanger sequencing confirmed that the *MYBPC3* c.787G>A variant is present exclusively in the proband (III-9), while all other tested individuals (II-4, III-9, II-5, III-10, II-1, II-3, and II-2) are wild-type.
